# The impact of implicit theories on resilience among Chinese nurses: The chain mediating effect of grit and meaning in life

**DOI:** 10.3389/fpsyg.2022.940138

**Published:** 2022-07-28

**Authors:** Yixun Tang, Changjiu He, Lanling Feng, Dongmei Wu, Xiaojun Zhou, Tao Li, Lina He, Qiao Cai, Yuchuan Yue

**Affiliations:** ^1^Department of Nursing, The Clinical Hospital of Chengdu Brain Science Institute, MOE Key Laboratory for Neuroinformation, University of Electronic Science and Technology of China, Chengdu, China; ^2^Department of Community Prevention and Control, The Clinical Hospital of Chengdu Brain Science Institute, MOE Key Laboratory for Neuroinformation, University of Electronic Science and Technology of China, Chengdu, China; ^3^The Clinical Hospital of Chengdu Brain Science Institute, MOE Key Laboratory for Neuroinformation, University of Electronic Science and Technology of China, Chengdu, China

**Keywords:** implicit theories, mindsets, grit, meaning in life, resilience, China, nurses

## Abstract

Implicit theories refer to assumptions people hold about different domains, also known as mindsets. There are two implicit theories on the malleability of one’s ability: entity theory and incremental theory. They constrain and regulate people’s understanding and responses to an individual’s behavior, leading to different social cognitive patterns and behavioral responses. Resilience is a positive adaptation in highly stressful situations that represents mechanisms for coping with and transcending difficult experiences, i.e., a person’s ability to successfully adapt to change, resist the adverse effects of stressors, avoid significant dysfunction, and be chronically affected by considered a protective factor for mental health. Although previous studies showed that individuals’ implicit theories are associated with resilience, this relationship has received little attention in the nursing population. It is unclear which variables may contribute to explaining the relationship between implicit theories and resilience. Therefore, the current study aims to deeply explore the relationship between implicit theories and the resilience of Chinese nurses. In addition, we also seek to demonstrate the chain mediating effects of grit and meaning in life on this relationship. We surveyed 709 Chinese nurses through online questionnaires using the self-made demographic questionnaire, the Implicit Theories Scale, the Short Grit Scale, the Meaning in Life Questionnaire, and the 10-item Connor-Davidson Resilience Scale. After controlling for demographic variables such as age, gender, educational background, marital status, professional title, and working years, the results reveal positive associations between Chinese nurses’ implicit theories and their resilience, and grit and meaning in life play a partial mediating role in this relationship, respectively. Furthermore, grit and meaning in life play a chain mediating role between implicit theories and resilience. These findings contribute to understanding the psychological impact mechanism of implicit theories on nurses’ resilience and provide a theoretical basis for nursing managers to formulate strategies to improve nurses’ psychological resilience.

## Introduction

Nursing is a stressful profession that often involves a high workload, shift working, lack of adequate attention to the nursing profession, low social support, conflict with physicians, bullying and violence, and dealing with death, patients, and their families, etc. (Chang et al., 2005; Mcneely, 2005; Lin et al., 2014). Huge pressure from work leads to various physical and mental health problems for nursing staff (Botha et al., 2015). The health of nurses is crucial to ensuring the quality and safety of nursing care. Resilience is an important condition for maintaining individual physical and mental health (Kim and Chang, 2022) and has been regarded as a stress-coping mechanism (Cooper et al., 2021). Resilience is one’s ability to bounce back or recover from adversity. In the literature, resilience has been described into three types: (a) Trait or competence definition considers psychological resilience as a good adaptation of individuals in response to adverse events and as a trait and skill. (b) Outcome definition results from the positive, developmental adaptation of individuals in high-risk environments. (c) Process definition, psychological resilience, is a good adaptation process of individuals in the face of adverse events when internal and external protection and risk factors interact. Although there is no uniform definition of psychological resilience in the current research, most definitions revolve around the two cores of “stressful situations” and “positive adaptation” (Fletcher and Sarkar, 2013). This study looks at resilience as a trait or ability that is dynamically changing. Impaired resilience will not only damage the health of nurses but also lead to burnout or turnover intention, which is also the main problem troubling nursing managers (Eckleberry-Hunt et al., 2009; Gillespie et al., 2009). Evidence suggests that the resilience can help nurses rejuvenate and cope with adversity to sustain survival in challenging work environments (Gillespie et al., 2009). Specifically, resilience can prevent or reduce a variety of adverse consequences caused by job stress, such as anxiety, depression, post-traumatic stress disorder, job burnout, and willingness to leave (Rees et al., 2015; Luceño-Moreno et al., 2020). At the same time, it can ensure nurses’ work engagement and improve patient outcomes (Lyu et al., 2020), which is a key factor to help nurses cope with workplace adversity. Previous studies have found that resilience protects against a variety of psychological and psychiatric disorders among nurses directly involved in the care of infected patients (Schreiber et al., 2019). Studies have shown that when faced with the same pressures, challenges, and complex emotional needs, highly resilient nurses are more likely to respond positively, adapt well, and succeed in growth and career development (Ablett and Jones, 2007; McDonald et al., 2016). Therefore, building the resilience of nurses is important for them to cope with workplace stress and maintain their careers.

### The relationship between implicit theories and resilience

Regarding measures to promote and enhance nurses’ psychological resilience, researchers have mainly conducted empirical studies on positive psychology and external support (including family, organization, and society). Still, there is very little research revealing the impact of individual mindsets on psychological resilience. It has been shown that people can simultaneously hold opposite explicit and implicit attitudes toward the same object (Briñol et al., 2006; Rydell and McConnell, 2006) and that much of our behavior is driven by implicit beliefs that are not controlled by direct rationality (Nosek et al., 2011). Researchers agree that implicit attitudes have a stronger impact on individual behavior than explicit attitudes (Greenwald et al., 2009). Implicit theories (or mindsets) refer to beliefs that people hold regarding whether abilities are fixed or variable, including incremental theory (or growth mindset) and entity theory (or fixed mindset; Dweck and Yeager, 2019). Previous studies have considered implicit theories as a unidimensional construct, with the incremental and entity theories resting on opposite extremes of a continuum (Molden and Dweck, 2006). Implicit theories are essential in social psychology. It believes that individuals’ different implicit beliefs will affect their understanding and judgment of information, especially information in adversity or unfavorable environments, and then form different goal motivations and attribution types. Different target motivations and attributions further lead to different coping responses. Individuals who support entity theory believe that the attributes of people or things are fixed and are not affected by individual efforts, motivations, or situations, and tend to adopt negative coping strategies; those who support incremental theory believe that attributes are dynamically malleable and can be changed by individual efforts or the situation, they often adopt active coping strategies. Thus, implicit theories constitute a meaningful framework for individuals to interpret, predict, and judge events related to their internal world and influence how they perceive and react to the world. Different implicit theories lead to different goal orientations, motivations for achievement, and responses to difficulties and frustrations.

In general, people who support the incremental theory have higher psychological adjustment and better functional adaptability than those who support the entity theory (Snyder et al., 2014). Entity theorists often blame their lack of ability to change when they encounter setbacks or difficulties and are prone to helpless tendencies, negative emotions, and behaviors. In contrast, incremental theorists focus more on environmental factors, strive to find new solutions to improve the current situation, exhibit positive emotions and behaviors (Liu et al., 2013), and are more likely to overcome personal frustrations to promote psychological resilience (Schroder, 2021). Research shows that individuals who support entity theory have a poor ability to adapt to public health challenges, while individuals who support incremental theory can better recover from public health crisis events (Park and Shapiro, 2021). Previous studies have confirmed that mindsets (implicit theories) can predict the resilience of adolescent students (Yeager and Dweck, 2012). Recent studies have explored the relationship between mindsets (implicit theories) and the psychological resilience of American College adults, and found that people with a growth mindset show better psychological resilience than those with a fixed mindset (Boullion et al., 2021). In conclusion, these findings suggest that people who support the incremental theory (growth mindset) have better resilience. However, in the nurse population, the relationship between implicit theories and resilience has not been verified. Therefore, based on previous evidence, we attempt to propose the following hypotheses:

*Hypothesis* 1: implicit theories positively predict nurses’ resilience.

### The mediating effect of grit

Grit refers to persistence and enthusiasm for long-term goals, and it is the inner motivation that drives individuals to continue to meet challenges. In the definition by Duckworth et al. (2007), grit has two elements: persistence of effort in the face of adversity (perseverance) and consistency of interest (passion for long-term goals). Multiple cross-sectional studies have shown that a growth mindset is positively associated with grit (Zhao et al., 2018; Zeng et al., 2019; Park et al., 2020). A growth mindset may modulate grit-related brain structures (Wang et al., 2018) and play an important role in developing grit levels in late adolescence. In a longitudinal study of adolescents, grit and growth mindset mutually predicted each other’s developmental trajectories and did not differ by gender, race, or socioeconomic status (Park et al., 2020). Duckworth (2016) proposed that a growth mindset may lead to grit. When dealing with difficult situations and failures, individuals with a growth mindset are more optimistic, and the more autonomous their goal motivation is, the more likely they are to make an effort to persevere and maintain interest in the activities they are currently engaged in (Ryan and Connell, 1989; Ryan and Deci, 2000). Individuals with a fixed mindset show more negative emotions when faced with difficulties, and their intrinsic motivation is to avoid difficulties and not to make efforts. Type of motivations can influence grit through pathways of both perseverance and passion (Zhao et al., 2018). Individuals with a growth mindset, who are good at developing their own strengths and strive to improve themselves, will show greater grit over time (Kannangara et al., 2018). Grit and resilience are concepts that are often used interchangeably. However, research shows that they are different structures, and there is often a significant relationship between them (Musso et al., 2019; Meyer et al., 2020). Several studies have shown that grit is positively correlated with resilience (Calo et al., 2019; Shakir et al., 2020). Researchers suggest that gritty individuals may develop mechanisms for coping with adversity to promote well-being and prevent psychopathology, allowing individuals to recover from negative events and thereby maintain resilience (Musso et al., 2019). From this, it can be seen that implicit theories can affect resilience by affecting grit. Based on these findings, we propose the hypothesis:

*Hypothesis* 2: Implicit theories affect nurses’ resilience through grit.

### The mediating effect of meaning in life

The meaning in life is that people comprehend and understand the meaning of their own life, and realize the goal and mission of their own life, including searching for meaning and experiencing meaning, which runs through people’s lives and plays a positive role in promoting individual development (Steger et al., 2006). Evidence shows that in studies of difficult situations and failures, individuals with a growth mindset display positive feelings and emotions, while those with a fixed mindset display negative emotions, such as stress, anxiety, and depression (King et al., 2006; Hicks and King, 2007, 2008). Positive emotions are strongly positively correlated with meaning in life (King et al., 2006; Chu et al., 2020), and inducing positive emotions can enhance meaning in life even after considering other theoretical sources of meaning, Such as religion, social belonging, and global cognitive focus (King et al., 2006; Hicks and King, 2007, 2008). Furthermore, there is a strong positive correlation between meaning in life and resilience. On the one hand, meaning in life can promote resilience (Ostafin and Proulx, 2020); on the other hand, resilience may be a predictor of meaning in life (Luceño-Moreno et al., 2020). Previous research has found that individuals with a higher sense of meaning in life experience better psychological states (Yela et al., 2020) and are more resilient in the face of setbacks or trauma (Du et al., 2017). Research conducted during the COVID-19 pandemic has also shown that meaning in life is positively associated with positive experiences and resilience (Yildirim et al., 2021). Thus, a meaningful life may be a protective factor for improving people’s resilience. Based on the above, the following hypothesis is proposed:

*Hypothesis* 3: Implicit theories affect nurses’ resilience through meaning in life.

### The chain mediating effect of grit and meaning in life

The process model of resilience points out that individuals will use various resources to maintain a balance of physical and mental when dealing with stressful events, and the use of resources depends on the internal psychological characteristics of individuals. Incremental theorists tend to have positive psychological qualities and goal motivation. When faced with difficulties, individuals with incremental theory are more optimistic, have clear goals, and believe that hard work can help people achieve success. They are more likely to try to persevere and maintain interest in the activities they are currently engaged in (Ryan and Connell, 1989; Ryan and Deci, 2000). In this process, the type of motivations can influence grit through pathways of both perseverance and passion (Zhao et al., 2018).

Grit is associated with a higher existence and search for meaning in life, and gritty people are more likely to realize that life has meaning and are more motivated to find meaning in life. Gritty people work hard and stick to their long-term goals, even when they may face associated obstacles and failures. Thus, the ability of grit to promote commitment to important life goals may be associated with a greater sense of meaning in life (Datu et al., 2018). The study found that grit can significantly predict meaning in the life of Chinese nurses and has a positive impact on nurses (Datu et al., 2018). A strong meaning in life promotes resilience by inhibiting uncertainty and accompanying pain (Ostafin and Proulx, 2020). Therefore, the following hypothesis is proposed:

*Hypothesis* 4: Implicit theories affect the resilience of nurses through grit and meaning in life.

Although research has shown that implicit theories are significantly associated with individual resilience, the existing literature is still unclear on how implicit theories affect nurses’ resilience, the purpose of developing the current model is to further understand the psychological impact mechanism of nurses’ implicit theories on psychological resilience and try to understand how other individual psychological variables affect the relationship between them.

## Materials and methods

### Participants and procedure

The study adopted a cross-sectional design using a web-based questionnaire. 756 nurses from different hospitals in Chengdu and Kunming in Southwest China were recruited, and 709 nurses completed the questionnaire (the remaining participants were excluded because of missing data fields), and the effective response rate reached 93.78%. All participants signed an online informed consent form and voluntarily participated in this study. Inclusion criteria were: (a) obtained a vocational qualification certificate from the People’s Republic of China; (b) at least 1-year experience in clinical nursing or clinical nursing management; (c) no previous or current mental illness or drug or alcohol dependence; (d) Have the skills to complete questionnaires online; and (e) be told to take part in the study. Maternity leave, sick leave, training nurses, and nurses with incomplete responses to the questionnaire were excluded.

The study received ethics approval from the Ethics Committee of the Chengdu fourth Hospital and the registration number of the Chinese Clinical Trial Registry is ChiCTR1900020715. Participants were recruited through online advertising. Before the investigation, online informed consent was obtained and responses were anonymous. All the information collected was stored securely. All questionnaires were self-rated, and participants filled them out separately.

### Measures

#### Demographic data

The self-made general information questionnaire was used to collect the socio-demographic characteristics, including the age, gender, educational background, marital status, professional title, and working years of the subjects. Table 1 describes the study sample’s basic demographic characteristics and basic information.

**Table 1:** Baseline characteristics and differences in the resilience score of nurses (*N* = 709).

Variables	Frequency (percentage)	Resilience scale (*M* ± SD)	*t/F*	*P*
Age(years)			4.212	0.006
< 30	319(44.99%)	36.57 ± 5.44		
≥ 30	277(39.07%)	37.64 ± 5.16		
≥ 40	94(13.26%)	38.64 ± 6.51		
≥ 50	19(2.68%)	36.37 ± 6.43		
Gender				
Male	66(9.3%)	38.49 ± 4.71	1.892	0.059
Female	643(90.7%)	37.13 ± 5.62		
Educational background				
Technical secondary school	37(5.22%)	33.92 ± 5.42	4.803	0.003
Junior college	270(38.08%)	37.37 ± 5.61		
Undergraduate	398(56.14%)	37.48 ± 5.47		
Master	4(0.56%)	37.75 ± 2.22		
Marital status				
Unmarried	198(27.93%)	35.91 ± 5.84	4.944	0.001
Married	480(67.70%)	37.71 ± 5.31		
Divorce	26(3.67%)	39.08 ± 5.95		
Widow	2(0.28%)	41.00 ± 4.24		
Other	3(0.42%)	34.33 ± 6.66		
Professional title				
No title	163(22.99%)	36.10 ± 5.87	4.403	0.004
Junior title	366(51.62%)	37.36 ± 5.15		
Intermediate title	170(23.98%)	37.95 ± 5.98		
Deputy senior professional title	10(1.41%)	40.40 ± 3.53		
Working years				
< 10 years	407(57.40%)	36.80 ± 5.22	4.852	0.008
10–19 years	192(27.08%)	37.44 ±5.52		
≥ 20 years	110(15.51%)	38.62 ± 6.54		

Age: 1 = < 30 years, 2 = ≥ 30 years, 3 = ≥ 40 years, 4 = ≥ 50 years; Gender: 1 = male, 2 = female; education background: 1 = technical secondary school, 2 = junior college, 3 = undergraduate, 4 = master; Marital status: 1 = unmarried, 2 = married, 3 = divorce, 4 = widow, 5 = other; Professional title: 1 = no title, 2 = junior title, 3 = intermediate title, 4 = deputy senior professional title; Working years: 1 = < 10 years, 2 = 10–19 years, 3 = ≥ 20 years.

#### Implicit theories

The Implicit Theories of Personality Scale was adapted for the present study (Levy et al., 1998). This eight-item scale contains four items for entity theory (fixed mindset; e.g., How a person is, reflects the most basic things about him and does not change much) and four for incremental theory (growth mindset; e.g., Everyone, no matter who they are, can dramatically change their basic traits). The higher the score, the more inclined to the incremental theory, and the lower the score, the more inclined to the entity theory. Participants rate their beliefs on the fixedness and malleability of their personality using a 6-point Likert scale from 1 = (strongly disagree) to 6 = (strongly agree). In this study, the Cronbach’s *α* coefficient of this scale was 0.796.

#### Grit

The self-reported Short Grit Scale (Grit-S) was developed and validated by Duckworth and Quinn (2009), including two subscales, namely consistency of interest (e.g., Setbacks do not make me discouraged) and perseverance of effort (e.g., No matter what, as soon as I start it, I’ll finish it). A total of eight items in the scale were included and used the 5-point Likert scale (from 1 = “not like me at all” to 5 = “very much like me”). The Grit-S of the Chinese version has good reliability which had been verified by multiple studies (Zhong et al., 2018; Luo et al., 2020). In this study, the Cronbach’s α of the total scale and subscales were 075, 0.68, and 0.72, respectively.

#### Meaning in life

The 10-item Meaning in Life Questionnaire (MLQ) was developed by Steger et al. (2006), including two subscales, the presence of meaning (e.g., I understand the meaning of my life) and the search for meaning (e.g., I am looking for a purpose or mission in my life). The Chinese version of MLQ was revised and verified by Wang (2013). Participants rated on a 7-point Likert response format (from 1 = “completely inconsistent” to 7 = “completely consistent”). In this study, the Cronbach’s *α* coefficient of the total scale was 0.872. The Cronbach’s *α* coefficients of the two subscales were 0.866 and 0.897, respectively.

#### Resilience

Connor-Davidson Resilience Scale (CD-RISC-10) was used to assess nurses’ resilience. The scale was modified from CD-RISC. The scale contained 10 items (e.g., I can adapt when things change), each of which was scored on a Likert 5-point scale, with 0 indicating never and 4 indicating always, and higher scores indicating higher levels of resilience. The Chinese version of CD-RISC-10 has good reliability and has been verified by many studies (Meng et al., 2019; Cheng et al., 2020). In this study, the scale had good reliability, with a Cronbach’s *α* of 0.927.

#### Control variables

According to the statistical analysis results in Table 1, current research takes the nurses’ age, gender, educational background, marital status, professional title, and working years of the subjects as the control variables.

### Statistical analysis

The study used SPSS 21.0 and the Process plug-in to analyze the data and used Process model 6 to test the chain mediation model. The measurement data were expressed by *M* ± SD, and the count data were expressed by frequency and percentage. Independent samples *t*-test or one-way ANOVA was used to analyze the impact of different demographic characteristics on resilience. Pearson’s correlation analysis was adopted among implicit theories, grit, meaning in life, and resilience. Process V3.5.2 Model 6 is used to test chain mediation and regression analysis, the bootstrap method was used to estimate the 95% confidence interval with 5,000 repeated sampling, and two-sided inspection level *α* = 0.05.

## Results

In the final valid sample, women accounted for 90.7% and men accounted for 9.3%. From the perspective of age distribution, the sample nurses are basically under 40 years old. In this study, 56.6% of the nurses had a bachelor’s degree or above. 67.7% of the nurses are married, most of them have low professional titles, mainly nurses with junior professional titles, and most of them work within 10 years (Table 1).

### Common method bias test

First, before data processing, Harman’s single-factor test was used (Podsakoff et al., 2003); if the one general factor accounts for more than 40% of the total variance, it indicates the presence of common method variance. In this study, there are seven factors with eigenvalues > 1, and the variation explained by the first factor is 32.73%, which is less than the critical criteria of 40%, indicating that the common method bias is not obvious.

### Descriptive statistics and correlation analysis

The statistical descriptions of the variables and the relationships between the variables are shown in Table 2, and the results show that the variables of nurses’ implicit theories, grit, meaning in life, and resilience were significantly and positively correlated. The relationship between variables supports the subsequent testing of the hypotheses.

**Table 2:** Descriptive statistics of various variables and associations between the variables (*N* = 709).

Variables	*M*	SD	1	2	3	4
1. Implicit theories	29.51	4.95	1			
2. Grit	27.03	4.14	0.520^**^	1		
3. Meaning in life	49.84	8.29	0.412^**^	0.463^**^	1	
4. Resilience	37.26	5.55	0.490^**^	0.553^**^	0.622^**^	1

** *p* < 0.01.

### The chain mediating analysis

**Figure 1:**
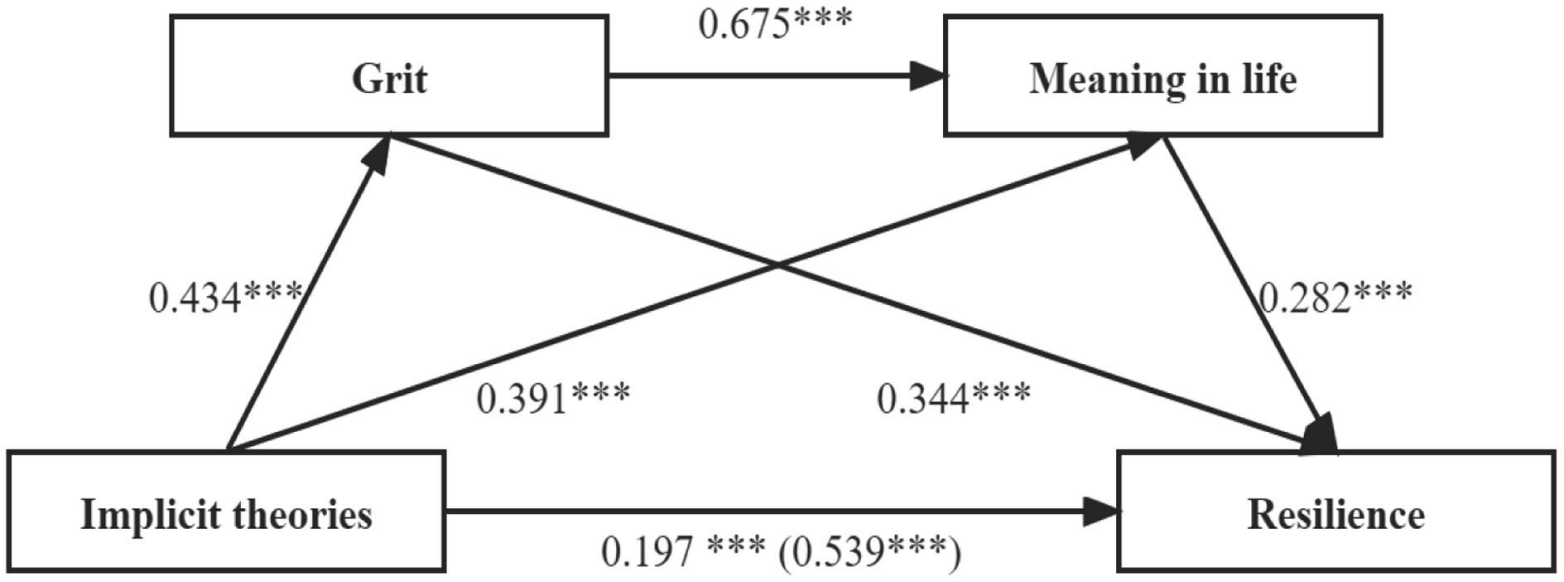
Model of the mediator role of grit and meaning in life in the relationship between implicit theories and resilience. ^***^p < 0.001, significant regression coefficient.

Model 6 in the SPSS macro program PROCESS3.5.2 developed by Hayes was used to analyze the mediating effects. After age, gender, educational background, marital status, professional title, and working years were controlled, the implicit theories were used as the independent variable, resilience as the dependent variable, and grit and meaning in life as mediating variables. The results of the regression analysis are shown in Table 3, the results of the mediation effect analysis are shown in Table 4, and the path relationship between the variables is shown in Figure 1.

**Table 3:** Regression analysis of the relationship between variables (*N* = 709).

Outcome variable	predictor variable	*β*	*t*	LLCI	ULCI	*p*	*R*²	*F*
Grit	Implicit theories	0.434	16.113	0.381	0.487	*p* < 0.001	0.280	39.103
Meaning in life	Implicit theories	0.391	6.110	0.265	0.517	*p* < 0.001	0.260	30.850
	Grit	0.675	8.801	0.524	0.825	*p* <0.001		
Resilience	Implicit theories	0.197	5.526	0.127	0.267	*p* < 0.001	0.515	82.732
	Grit	0.344	7.862	0.258	0.430	*p* < 0.001		
	Meaning in life	0.282	13.750	0.241	0.322	*p* < 0.001		
Resilience	Implicit theories	0.539	14.833	0.468	0.611	*p* < 0.001	0.270	37.087

**Table 4:** The chain mediating effect of grit and meaning in life.

Path	Effect	Boot SE	Boot LLCI	Boot ULCI	Relative effect
Total effect	0.539	0.036	0.468	0.611	100%
Direct effect	0.197	0.035	0.127	0.267	36.55%
Total indirect effect	0.342	0.035	0.277	0.415	63.45%
Path 1 implicit theories → grit → resilience	0.149	0.023	0.106	0.197	27.64%
Path 2 Implicit theories → meaning in life → resilience	0.110	0.027	0.062	0.169	20.41%
Path 3 Implicit theories → grit → meaning in life → resilience	0.083	0.013	0.057	0.110	15.40%
C1 (Ind1 minus Ind2)	0.039	0.039	-0.041	0.113	
C2 (Ind1 minus Ind3)	0.067	0.027	0.014	0.122	
C3 (Ind2 minus Ind3)	0.027	0.029	-0.025	0.089	

The results showed the total effect (*β* = 0.539, *t* = 14.833, *p* < 0.001) and the direct effect (*β* = 0.197, *t* = 5.526, *p* < 0.001) of implicit theories on the resilience were both significant (Tables 3, 4). The regression analysis results are shown in Table 3; after demographic variables such as age, gender, educational background, marital status, professional title, and working years were controlled, implicit theories could significantly positively predict resilience (*β* = 0.197, *p* < 0.001); implicit theories could significantly positively predict grit (*β* = 0.434, *p* < 0.001) and meaning in life (*β* = 0.391, *p* < 0.001); grit could significantly positively predict both the meaning in life (*β* = 0.675, *p* < 0.001) and resilience (*β* = 0.344, *p* < 0.001); and meaning in life could significantly positively predict resilience (*β* = 0.282, *p* < 0.001).

In the test for mediating effects, the Bootstrap method was used to calculate 95% confidence intervals for each of the 5,000 repeated draws. Results of the mediating effect analysis in Table 4 showed that Bootstrap’s 95% CI of total indirect effect did not contain 0 [Bootstrap 95% CI: 0.277, 0.415], accounting for 63.45% of the total effect. The impact of implicit theories on the resilience of nurse groups is carried out through three mediating pathways. The first path is “implicit theories → grit → resilience,” the effect value is 0.149, the Bootstrap 95% CI is 0.106 and 0.197, excluding 0, and mediating effect is significant. The second path is “implicit theories → meaning in life → resilience,” the effect value is 0.110, and the Bootstrap 95% CI is 0.062 and 0.169, excluding 0, and mediating effect is significant. The third path is “implicit theories → grit → meaning in life → resilience,” the effect value is 0.083, and the Bootstrap 95% CI is 0.057 and 0.110, excluding 0, chain multi-mediating effect is significant. In conclusion, grit and meaning in life not only play an independent mediating role between implicit theories and resilience but also play a chain mediating role. The Chain mediating model is shown in Figure 1.

A pairwise comparison of the different indirect effects paths was adopted to verify whether these paths were significantly different. The results showed that Comparison 2 [Bootstrap 95% CI:0.014,0.122] was significant, except Comparison 1 [Bootstrap 95% CI: −0.041,0.113] and 3 [Bootstrap 95% CI: −0.025,0.089].

## Discussion

The results of this study show that implicit theories positively predict the level of resilience of nurses, confirming Hypothesis 1, which is similar to previous findings (Yeager and Dweck, 2012; Boullion et al., 2021). This study indicates that nurses who are more inclined to incremental theory show higher resilience in the face of challenges or adversity than nurses who adopt entity theory. Previous research has suggested that implicit theories may influence individuals’ responses and reactions to interpersonal conflict (Chan et al., 2014). Researchers found that individuals’ beliefs that personality was malleable influenced their use of conflict management strategies in romantic relationships (Kammrath and Dweck, 2006). Individuals who support the incremental theory often face and tolerate anxiety, setbacks, and disappointments in healthy and adaptive ways that increase resilience (Schroder, 2021). Research has shown that growth mindset interventions in the personality domain can also improve individuals’ stress coping styles, reduce stress levels, improve cardiovascular and circulatory system function, and enhance their mental resilience, enabling them to cope positively when they experience negative emotions (Yeager et al., 2016). The reason why nurses with incremental theory can show high resilience maybe they can always face various occupational pressures with optimism, strive to improve their abilities, adopt positive coping strategies to protect themselves, and “recover” from negative events. When faced with professional challenges, those nurses with entity theory believe that personal attributes cannot be changed, and generally adopt avoidance behaviors for unsure things. As time goes on, their coping ability becomes worse and manifests as lower resilience. To our knowledge, this is the first report that implicit theories positively predict nurses’ resilience levels. This inspires us to strengthen the cognitive reconstruction training of nurses, cultivate nurses’ growth mindset, and shape the belief that ability can be changed, which will help improve nurses’ ability to cope with stress or challenges, better adapt to various negative events, and maintain good working status.

The results of the mediation effect analysis show that grit plays a partial mediating role between implicit theories and resilience, which confirms Hypothesis 2 of this study. After controlling for demographic variables such as age, gender, educational background, job title, marital status, and years of employment, the results show that incremental theory predicts nurses’ higher grit, which, in turn, positively affects nurses’ resilience, which is consistent with other research findings (Calo et al., 2019). A meta-analysis showed that implicit theories predict the process of self-regulation, while the process of self-regulation predicts perseverance for effort (Burnette et al., 2013). When nurses with incremental theory deal with stressful events, they generally have clear goals and motivations, are able to view the problem in an integrated manner, adjust and refine their goals in a timely manner by analyzing the problem, playing to their strengths, and respond positively, while being good at managing their emotions and maintaining an optimistic and positive mindset. When dealing with difficult situations and failures, individuals with a growth mindset are more optimistic, and the more autonomous their goal motivation is, the more likely they are to make an effort to persevere and maintain interest in the activities they are currently engaged in (Ryan and Connell, 1989; Ryan and Deci, 2000). Individuals with a fixed mindset show more negative emotions when faced with difficulties, and their intrinsic motivation is to avoid difficulties and not to make efforts. Positive attitudes and motivation motivate nurses to work hard to solve current difficulties, never give up easily, and believe that they can achieve their goals. As goals are achieved, the nurse’s grit attribute will be further strengthened. A gritty personality enables individuals to have more positive emotions (Datu and Restubog, 2020), and positive emotions will broaden nurses’ thinking and attention, enabling them to fully mobilize the power of internal protective factors and external support in the face of stressful events, and rebound from negative events (Musumari et al., 2018; Datu and Restubog, 2020).

The mediation effect analysis showed that meaning in life also plays a partial mediating role between implicit theories and resilience, confirming Hypothesis 3 of this study. The results showed that for nurses who were more inclined to the incremental theory, the perception of meaning in life was stronger, which, in turn, has a positive effect on nurse resilience. Implicit theories affect the judgment of self-concept by affecting the connection between negative feedback information and individuals (Howe and Dweck, 2016). Incremental theory can help individuals produce a positive self-concept and experience more positive emotions. A stable and clear self-concept enables individuals to more accurately understand themselves, the world, and themselves in this world, to understand their own life experiences or experiences from a longer-term goal and a higher level, and to grasp the meaning in various life events (Shin et al., 2016). Positive emotions activate the individual’s general knowledge framework, which may induce individuals to extract more information related to meaning, thereby enhancing the sense of meaning. When nurses deal with professional pressure, such as medical disputes and violence in medical places, entity theorists tend to avoid, negatively evaluate, and question themselves. Negative feedback information will weaken nurses’ interest and motivation for self-development, which is an important part of realizing the meaning of life. On the contrary, incremental theorists are often able to actively interpret the events they encounter, make positive evaluations of the status quo and their own abilities, look at all life experiences from a long-term perspective, and dig out the meaning of life to support their own development. Meaning in life protects against stressor-related subjective distress and repetitive negative thinking promotes psychological resilience through inhibiting uncertainty and concomitant distress (Ostafin and Proulx, 2020). Research during the COVID-19 pandemic has shown that meaning in life helps people cope with stressors and improves their mental health and resilience by helping them move beyond survival (Arslan et al., 2020; Yildirim et al., 2021). It is obvious that nurses with a stronger sense of meaning are able to find positive meanings in difficult situations and mobilize their inner protective factors to recover from negative emotions by relying on their own inner psychological energy when facing difficult situations.

The results show that grit and meaning in life play a chain mediating role between implicit theories and resilience, confirming Hypothesis 4 of this study, which is also an important finding of this study. That is to say, the impact of implicit theories on nurses’ resilience can be achieved through the mediating effect of grit and meaning in life. Facing various workplace pressures, nurses with incremental theory always maintain a positive and optimistic attitude, have positive goal motivation, and firmly believe that unremitting efforts can overcome all difficulties, in the process of constantly overcoming difficulties, they gradually formed tenacious courage, namely grit. Gritty nurses will not give up easily when they encounter difficulties. They are full of hope for the future and work hard to solve problems. During this process, nurses have higher expectations for themselves, life, and their nursing career. The sense of achievement and value of the profession allow nurses to experience more meaning. Gritty nurses are good at finding resources to help themselves grow, and research shows that nurses with high grit can improve their meaning in life and quality of life through social support (Yang and Wu, 2021), which further supports the results of this study. Having a clear sense of purpose and meaning in life can help people overcome emotional distress (such as anxiety, and depression) caused by adversity, buffer stress, and enhance resilience (Arslan et al., 2020; Yildirim et al., 2021). The more nurses perceive the meaning of life, the clearer their self-awareness, the stronger their motivation to pursue goals, the more likely they are to experience a sense of accomplishment and satisfaction in their daily lives, and face stress or setbacks with a more positive attitude, thus demonstrating high levels of resilience.

The results also show that the mediating path of grit has the greatest impact on the overall mediating effect, indicating that the impact of implicit theories on resilience is largely played by grit. Grit is a positive psychological quality, an important condition for predicting people’s success in different fields such as school, career, family, etc., and its predictive effect on success even exceeds intelligence and creativity (Tyer-Viola, 2019). Nurses with grit may not be as smart as others, but the personality of being gritty makes them passionate about nursing, and constantly improve their abilities to establish effective coping mechanisms, often most successful in coping with workplace stress. Thus, grit plays an important role in the psychological mechanisms associated with implicit theories and resilience.

This study deeply explores the psychological influence mechanism of implicit theories on Chinese nurses’ resilience, and that grit and meaning in life play an important role in promoting Chinese nurses’ resilience. This result extends previous research on implicit theories and individual resilience. The findings explain the antecedents that affect nurses’ resilience from a social cognitive perspective. Our study has important implications for interventions designed to improve nurses’ resilience. Our findings underscore that incremental theory can provide a powerful impetus for nurses to improve resilience. Therefore, medical institutions, nursing organizations, and nursing managers should realize the key role of implicit theories in nurses’ coping with stress or challenges, and create a work atmosphere that emphasizes that efforts can change the status quo and achieve success. For those nurses with entity theory, nursing managers should encourage them to be proactive, using peer support (Sheffler and Cheung, 2020) and role model learning (Du et al., 2021) to promote their incremental theory. In addition, cultivating nurses’ willpower, the meaning of life, and other positive psychological qualities is also conducive to promoting nurses’ adversity adaptability.

## Limitations

There are some deficiencies in this study: First of all, this study adopts a cross-sectional study, and cannot draw a rigorous causal relationship on the relationship of each variable; Second, the use of nurses in a certain region of China as the research object in this study may have some impact on the external applicability of the results; Third, this study did not analyze and report the interaction between grit and meaning in life. Finally, the data collection of each variable in this study rely on an online self-rating scale, and the resulting recall bias may have a certain impact on the accuracy of the data.

## Conclusion

In conclusion, this study investigates how Chinese nurses’ implicit theories influence resilience. Specifically, we found that implicit theories significantly positively predicted resilience and verified the mediating role of grit and meaning in life in this relationship. The results also confirmed a chain mediation model between implicit theories, grit, meaning in life, and resilience.

## Data availability statement

The raw data supporting the conclusions of this article will be made available by the authors, without undue reservation.

## Ethics statement

The studies involving human participants were reviewed and approved by the Research Ethics Committee of Chengdu 4th Hospital. The patients/participants provided their written informed consent to participate in this study.

## Author contributions

All authors contributed to the design of the study, the distribution of questionnaires, the collection of data, the writing of the manuscript, and the submission of contributions. All authors contributed to the article and approved the submitted version.

## Funding

This work was supported by the Sichuan Science and Technology Program (grant 2018JY0306) and the National Natural Science Foundation of China (grant 82001444).

## Conflict of interest

The authors declare that the research was conducted in the absence of any commercial or financial relationships that could be construed as a potential conflict of interest.

## Publisher’s note

All claims expressed in this article are solely those of the authors and do not necessarily represent those of their affiliated organizations, or those of the publisher, the editors and the reviewers. Any product that may be evaluated in this article, or claim that may be made by its manufacturer, is not guaranteed or endorsed by the publisher.
